# Low-dose ionizing radiation and the exposure–lag response: protocol for a prospective cohort study on The Health Effects of Chongqing Occupational Radiation Workers

**DOI:** 10.3389/fpubh.2025.1531546

**Published:** 2025-01-27

**Authors:** Xiao-Ling Qin, Qiang Huang, Han-Wen Zhang, Yi Zeng, Xian-Shu Lin, Xiao-Yuan Fan, Jun Diao, Cheng-Zhi Chen, Shu-Qun Cheng, Fang Yuan, Jun-Lin He, Wei Li, Yin-Yin Xia

**Affiliations:** ^1^School of Public Health, Chongqing Medical University, Chongqing, China; ^2^Chongqing Center for Disease Control and Prevention, Chongqing, China; ^3^Department of Obstetrics, The First Affiliated Hospital of Chongqing Medical University, Chongqing, China; ^4^Chongqing Jiulongpo District Center for Disease Control and Prevention, Chongqing, China

**Keywords:** radiation workers, low-dose ionizing radiation, exposure–lag response, cohort, distributed lag non-linear model

## Abstract

**Introduction:**

Although the effects of ionizing radiation on radiation workers have been extensively studied in China, no prospective cohort study has been conducted in Chongqing. Furthermore, previous cohorts have not provided a broad-gauge assessment of the temporal relationship between low-dose occupational radiation exposure and the risk of health outcomes.

**Methods:**

A prospective cohort study will be carried out focusing on radiation workers in Chongqing. Health examination outcomes and radiation dose monitoring data will be collected and analyzed using the distributed lag non-linear model (DLNM) combined with generalized additive model (GAM) or generalized linear model (GLM) to evaluate the exposure-lag response relationship.

**Discussion:**

Our study will enhance our understanding of the exposure-lag response association between occupational radiation exposure and the health of radiation workers based on DLNM.

**Clinical trial registration:**

Chinese Clinical Trials Registry, ChiCTR2400081804.

## 1 Introduction

Ionizing radiation is extensively applied in medicine, industry, and other fields. Ionizing radiation can induce cell death, impair the integrity of organs and tissues, and impair their function, which are collectively known as the biological effects, including deterministic and stochastic effects (1). The severity of deterministic effects increases as absorbed doses rise, and a threshold dose was required to observe injuries (1). Early effects include skin burns, reduced blood cell levels, and other symptoms. Later-occurring effects include cataracts, necrosis, hypothyroidism, and cardiovascular disorders (1). Absorption of ionizing radiation energy by genetic material in cells causes DNA damage, leading to cell death, chromosomal aberrations, and gene mutations (2), developing genetic diseases in the offspring of an irradiated individual or cancer, defined as stochastic effects (3). In addition, ionizing radiation exposure can damage liver and kidney function, and a few studies have proved this (4, 5).

With the continuous development of radiodiagnosis and therapy technology, the number of radiation workers in the medical field has significantly increased, around 600,000 radiation workers in China, with around 400,000 of these individuals engaged in medical occupations (6). Despite the use of appropriate personal protective equipment by radiation workers, they remain inevitably subject to chronic low-dose occupational radiation (7). Long-term exposure to low-dose ionizing radiation alter immune fitness, and increased risks of cardiovascular diseases and solid cancer (8–10). Among these health outcomes, cancer remains the primary focus of most epidemiological and cohort studies examining occupational radiation exposure (11, 12). The effects of ionizing radiation on radiation workers have been extensively studied in at least eight countries (Canada, the United Kingdom, Russia, Korea, Finland, France, Germany, Sydney, and the United States) (9, 13–30). Some cohort studies conducted nationally and through international collaborations, were characterized by their large scale and extended follow-up periods, for example, in a collaborative cohort of 15 countries, the study population comprises up to 407,391 individuals (21), in another study investigating the risk of malignant skin tumors among radiation workers, the follow-up period extended to about 70 years (30). However, the exposure-lag response associations between chronic low-dose ionizing radiation exposure and various adverse health outcomes have been not well-studied, with the exception of cancer (9, 31).

The exposure-lag response was delineated by Gasparrini regarding the temporal relationships between exposure and the risk of a health outcome (32). Quantitative exposure studies often use cumulative metrics, but lack detailed time-varying intensity data for the low-dose ionizing radiation exposure, while duration metrics omit intensity information (33). The exposure-lag response could explain the relative importance of intensity, duration, and timing of the low-dose ionizing radiation exposure in relation to various adverse health outcomes (10, 31–33). To address the exposure-lag response, we proposed to establish a prospective cohort in Chongqing characterized by personal radiation exposure dose, long follow-up duration, and relative various adverse health outcomes.

## 2 Methods and analysis

### 2.1 Study population and design

The THECORW study is a prospective cohort study. A total of 825 radiation workers will be recruited in Chongqing from 2024 to 2044. The study aims to include radiation workers in Chongqing. In accordance with the regulations on occupational health management of radiation workers issued by the National Health Commission of China, occupational health management is mandated for all radiation workers nationwide (34). Consequently, all radiation work units in Chongqing are required to organize regular occupational health examinations for radiation workers, with the interval between two consecutive examinations not exceeding 2 years (34). Radiation workers registered in the Chongqing CDC personal dose monitoring system will receive information about the study during their annual occupational health examinations from our investigators. Workers who consent to participate will provide written informed consent before enrollment. Those meeting the inclusion criteria will be enrolled in the study. Following enrollment, their personal dose monitoring data from the Chongqing CDC personal dose monitoring system will be integrated with health data from National Radiation Health Information Platform via personal identification numbers (35). Participants will retain the right to withdraw from the study at any time and for any reason without any consequences.

### 2.2 Inclusion and exclusion criteria

Included in the study will be radiation workers who are: (1) consistently wear dose dosimeters; (2) regular occupational health examination at the Chongqing Center for Disease Control and Prevention (CDC); (3) over 18 years old; (4) no communication barriers (34, 36). Excluded from the study will be those who are with a history of chromosomal aberrations, micronucleus abnormalities, peripheral blood abnormalities and blood-related diseases, abnormal liver and kidney function, cancer, history of abnormal thyroid hormones and thyroid-related diseases, and cataract and glaucoma (34, 36).

### 2.3 Sample size calculation

Radiation exposure damages hematopoietic tissues, which is characteristic of occupational exposure. It has been proven that the hematological system, including mature functional cells, is susceptible to ionizing radiation (36). Ionizing radiation can cause early damage to the hematological system, therefore, blood cell counts are commonly used to assess its effects, a method widely used in the field (36).

The complete blood count served as the basis for sample size calculation. A sample size of 825 is considered sufficient to allow studies of the impact of radiation on human health. For example, according to references, the white blood cell count is commonly used to assess the effects of radiation on the health of a population (3, 4). The average white blood cell count for the population in the Chinese radiation group is (6.34 ± 1.40)*10 (9)/L, and in the normal population group is (6.59 ± 1.39)*10^9^/L.^5^ For a type I error of 0.05, a type II error of 0.1, and a power of 0.9, the sample size required is 660. Considering a 20% loss in follow-up (37, 38), the sample size increases to 825. Therefore, a recruitment sample of 825 radiation-exposed individuals will have sufficient power for the study. The sample size was estimated using G*Power software version 3.1 (Heinrich-Heine-Universität Düsseldorf, Düsseldorf, Germany) (39).

### 2.4 Dosimetry data

Radiation dose data were collected based on the Chongqing CDC’s personal dose monitoring system, which integrates various functions, including sample collection and distribution, dose allocation and input, report generation, annual dose statistics and inquiries, and charge management (40). In addition, it provides functions supporting the entire monitoring process, such as new system management, automatic generation of periodic test reports, and annual dose reports (40).

The annual radiation exposure dose for workers is less than the threshold (20 mSv), consequently, the individual dose equivalent Hp (10) was used to evaluate the effective occupational exposure dose (6, 41). The annual effective dosage is defined as the cumulative dose equivalent over four successive monitoring periods (3 months each) (41).

The effective dose (E) was calculated using the following equation: where W_T_ represents the weighting factor of each tissue, W_T_ is the tissue weight factor of the exposed organ or tissue, and H_T_ is the equivalent dose of the main exposed organ or tissue (42).

$$E=\underset{T}{\sum }W_{T}\cdot H_{T}$$

Nuclear medicine and interventional radiology workers were required to wear two personal dosimeters, both inside and outside lead clothing, to estimate the radiation dose (42). The following equation was used to calculate the effective dose of external radiation: *α* and *β* are the coefficients (The coefficients are different with or without thyroid shielding), H_m_ is the Hp (10) measured by a personal dosimeter worn inside the lead apron (43), H_n_ is the Hp (10) measured by a personal dosimeter worn on the outer collar of a lead apron (42).

$$E=\alpha \;H_{m}+\beta \;H_{n}$$

The collective effective dose denotes the sum of the effective doses for all radiation workers, S represents the collective effective dose, X_i_ is the effective dose, n is the total number of workers (44–46), the equation as follows:

$$S=\overset{n}{\underset{i=1}{\sum }}\;X_{i}$$

The organ dose estimation refers to the International Commission on Radiological Protection (ICRP) 116 organ dose conversion factor and irradiation geometry factor (47, 48). The equation is as follows: D_T_ is tissue or organ dose (Gray); H_p_(d) is badge dose measurement when calibrated as personal dose equivalent (Sv or rem); K_a_ is air kerma (Gray) (49).

$$D_{T}=H_{p}\left(d\right)\left[\;\left(\frac{D_{T}}{K_{a}}/\frac{H_{p}\left(d\right)}{K_{a}}\right)\;\right]$$

Estimation of eye lens dose: D_L_ present absorbed dose to the eye lens (mGy); f _(q):_ conversion coefficient from personal dose equivalent to absorbed dose of eye lens; H_p_(d): personal dose equivalent (mSv). H_P_ (3) is the personal dose equivalent at a depth of 3 mm in soft tissue and is used for measuring lens dose (50, 51).

$$D_{L}=f_{q}\;Hp_{\left(d\right)}$$

### 2.5 Statistical analysis

Health examination outcomes and individual dose monitoring data will be collected from Chongqing CDC personal dose monitoring system and National Radiation Health Information Platform (35, 40). Data quality will be assessed using the “dataquieR” package in R software, following the framework proposed by Schmidt et al. (52, 53). If radiation dose data is missing, we can use the notional dose to supplement the missing data, which represented the average dose for the same occupation during that year (54). The exposure–lag–response association between radiation dose and health outcomes will be evaluated using a distributed lag non-linear model (DLNM) combined with either a generalized linear model (GLM) or multiple generalized additive models (GAM) (55). For certain outcomes, if necessary, a nested case–control design will be applied, with controls time-matched to cases to ensure a comparable number of total person-time observations. A cross-basis function in the DLNM, utilizing natural spline basis functions, will be employed to construct a two-dimensional matrix that integrates radiation dose and lag time, thereby enabling a detailed analysis of the lag structure (55). For continuous health outcomes, a Gaussian distribution will be used as the link function; for count outcomes, a Poisson distribution will be applied; and for categorical outcomes, a binomial distribution will be employed in the GLM framework. Given the non-linear relationship between radiation dose and health outcomes, GAM will be applied to assess potential dose–response relationships. For non-linear relationships, piecewise linear spline models will classify participants into different exposure categories based on radiation exposure thresholds and will use these categories to assess linear dose–response relationships. Several sensitivity analyses were conducted to assess the robustness of the results. First, GAM or GLM analyses will be rerun after excluding cases with missing data for both controls and cases. Second, to account for potential interaction effects, interaction models were fitted by including variables such as exposure dose levels, age, gender, and occupational category in relation to health outcomes. Third, if significant interaction effects were identified, stratified analyses were performed, such as by occupational category or exposure dose levels, to explore these effects further. All analyses were carried out using R software (version 4.4.1). For normally distributed data, continuous variables are presented as mean ± standard deviation (SD); skewed data are presented as median or interquartile range (IQR). *p* values <0.05 were considered as statistically significant.

### 2.6 Baseline information

The basic characteristics of the study population in 2016 are presented in Table 1.

**Table 1:** Characteristics and health outcomes of radiation workers in Chongqing, 2016.

	Mean ± SD / M[IQR]/ n (%)
Age	37.00 [31.25, 47.00]
Gender *n* (%)	
Male	30 (60)
Female	20 (40)
Education *n* (%)	
Associate degree or below	7 (14)
Undergraduate	37 (74)
Master’s degree	6 (12)
Marital status *n* (%)	
Unmarried	18 (36)
Married	32 (64)
White blood cells (10^9^/L)	5.72 ± 1.46
Red blood cells (10^12^/L)	4.88 ± 0.65
Hemoglobin (g/L)	142.90 ± 21.40
Platelets (10^9^/L)	214.32 ± 58.01
Hematocrit (%)	42.77 ± 5.63
Mean corpuscular hemoglobin (pg)	30.20 [28.65, 30.98]
Alanine transaminase (U/L)	19.75 [15.90, 29.38]
Blood urea nitrogen (mmol/L)	5.12 ± 1.35
Creatinine (μmol/L)	58.05 [51.95, 72.90]
Uric acid (umol/L)	235.51 ± 51.81
Gamma-glutamyl transferase (U/L)	17.00 [13.00, 26.50]
Total bilirubin (μmol/L)	10.02 [6.93, 12.97]
Direct bilirubin (μmol/L)	3.25 [2.42, 4.18]
Alkaline phosphatase (U/L)	74.50 [57.25, 93.50]
Fasting blood glucose (mmol/L)	4.70 [4.40, 5.10]
Proteinuria *n* (%)	
Positive	3 (6)
Hematuria *n* (%)	
Positive	5 (10)
Audiometry *n* (%)	
>25 dB	0 (0)
Triiodothyronine (T3)	1.29 [1.12, 1.58]
Thyroxine (T4)	105.92 ± 16.59
Thyroid-stimulating hormone (TSH)	1.44 [0.80, 1.78]
Lens opacity *n* (%)	
Abnormal	2(4)
Dermatological assessments *n* (%)	
Abnormal	0 (0)
Micronucleus rate *n* (%)	
2‰	1 (2)
Chromosomal aberrations rate *n* (%)	
1%	1 (2)

IQR, interquartile range; SD, standard deviation.

**Figure 1:**
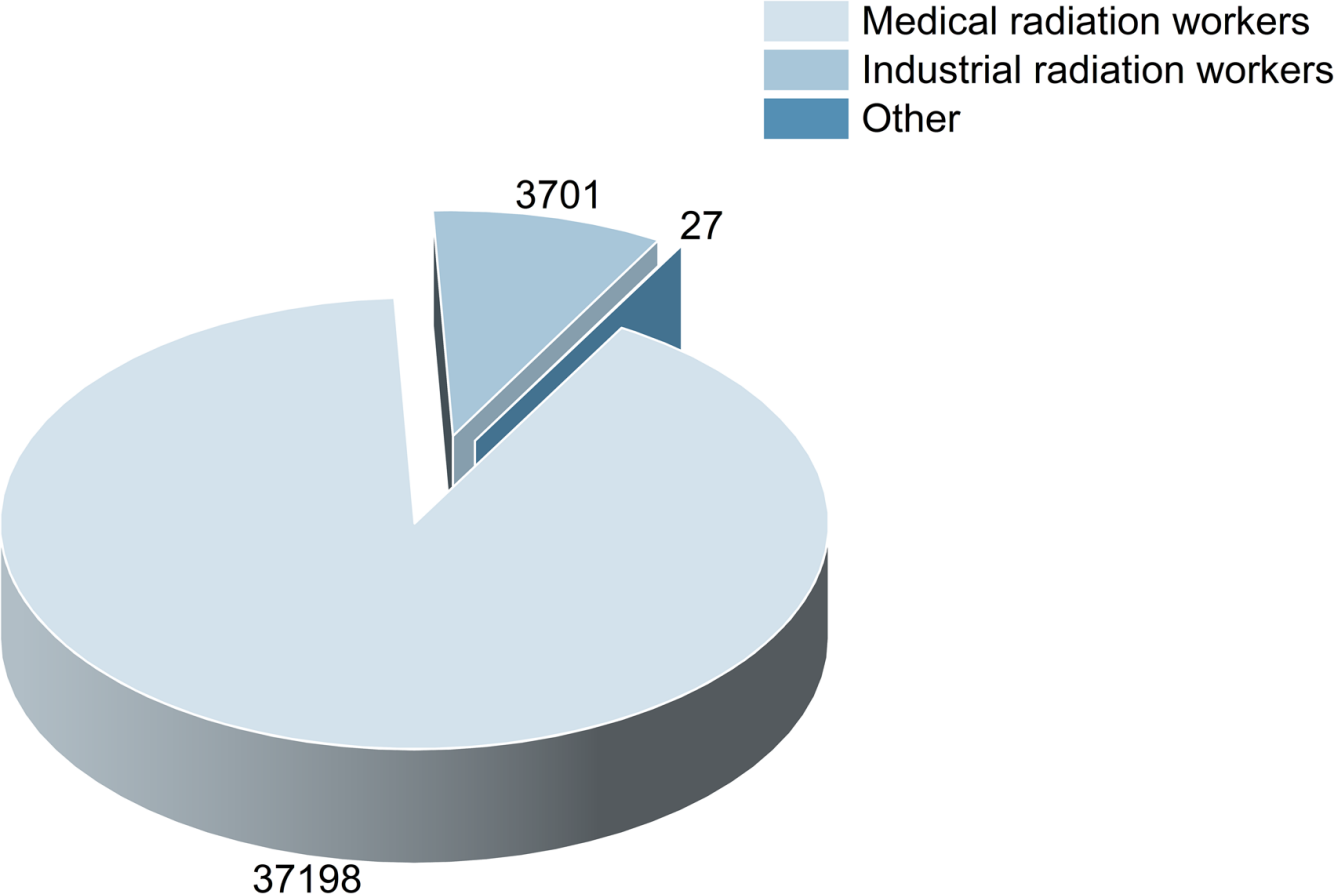
Distribution of Chongqing radiation workers according to occupation from 2016 to 2020.

According to the national standard GBZ 128–2019 (42), the target population includes medical radiation workers, industrial radiation workers, and others, excluding veterinary medicine. The distribution of workers across these three occupational categories in 2016–2020 is presented in Figure 1.

**Figure 2:**
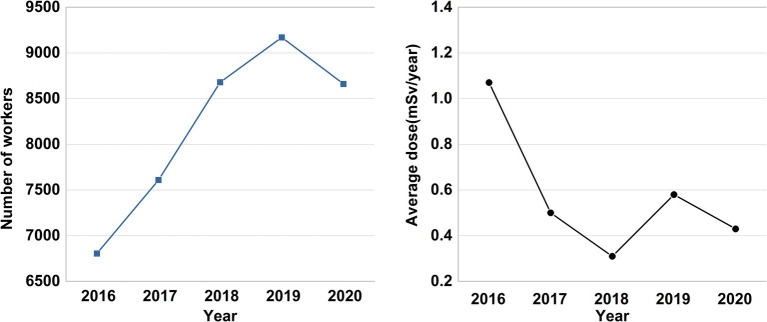
The annual number of radiation workers and their average annual effective dose in Chongqing from 2016 to 2020.

Although the number of industrial applications was small, the average annual individual effective dose was the highest (0.74 mSv). The average annual effective dose for radiation workers was 0.56 mSv in the past 5 years (6), below the worldwide average annual effective dose of 2.4 mSv from natural radiation sources, and far below the Chinese national standard personal dose limit of 20 mSv/ year (56, 57). The annual number of workers and their average annual effective dose are presented in Figure 2. Radiation workers in Chongqing have been exposed to low-dose ionizing radiation, with exposure levels exhibiting an overall downward trend over the past 5 years.

**Figure 3:**
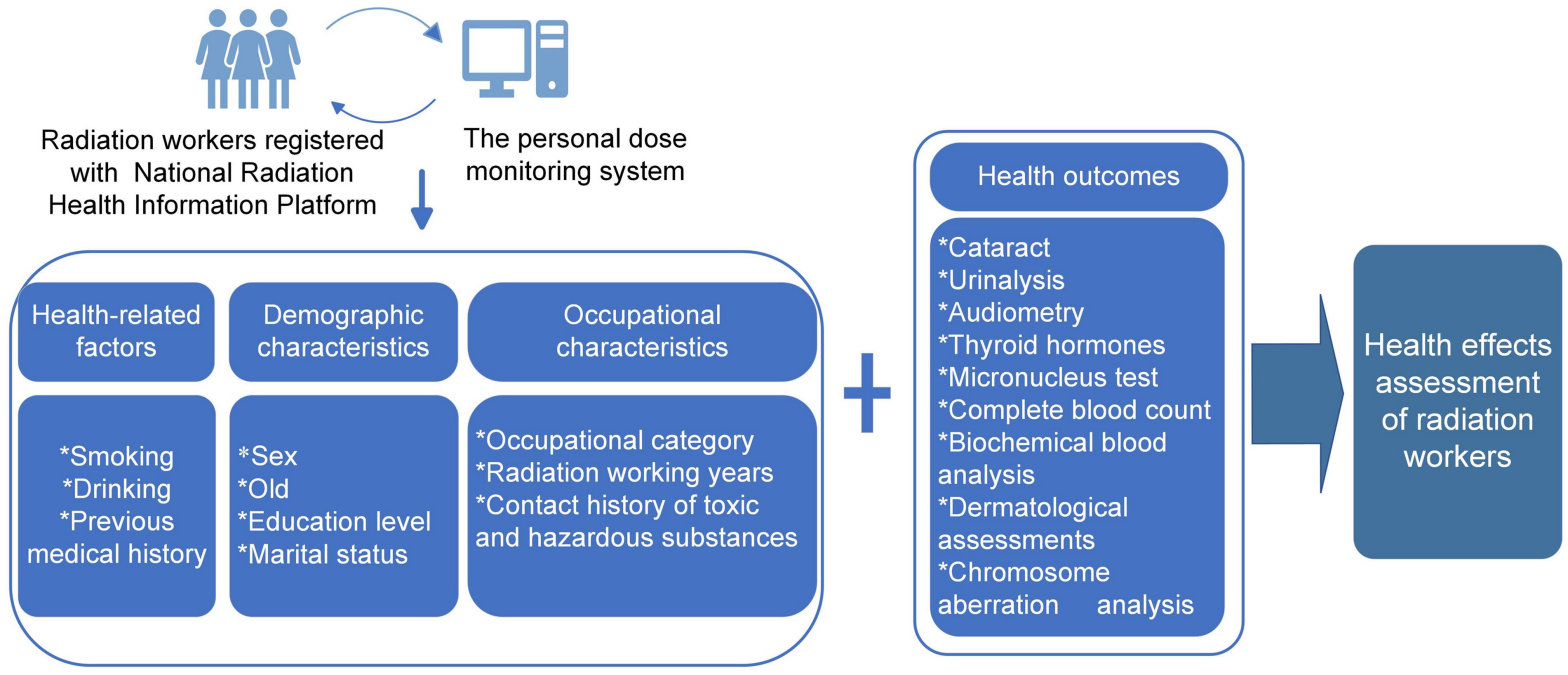
Flow chart of study design.

Detailed information will be collected from Chongqing CDC personal dose monitoring system, including workers’ age, sex, marital status, education level, previous medical history, smoking, drinking, contact history of toxic and hazardous substances, occupational category, radiation working years (40, 58). The study design is presented in Figure 3.

## 3 Health outcomes

Radiation workers undergo routine health examinations at the Chongqing CDC, health examination outcomes will be collected from the National Radiation Health Information Platform (35). These evaluations encompass a comprehensive set of assessments, including complete blood count, biochemical blood analysis, urinalysis, audiometry, thyroid function tests, ophthalmic examinations, dermatological assessments, micronucleus analysis, and chromosome aberration analysis, among them, the complete blood count is the primary health outcome. All examinations were conducted by the Health Examinations Department. The outcomes of these health evaluations are monitored to identify potential radiation-related health risks.

### 3.1 Primary health outcomes

#### 3.1.1 Complete blood count

After fasting for at least 8 h, blood samples were collected into EDTA anticoagulant tubes, and blood collection time and volume should be recorded. Then, hematological parameters were analyzed by an automated blood cell analyzer (SYSMEX, XS-1000, Japan) (36).

### 3.2 Secondary health outcomes

#### 3.2.1 Thyroid hormones

Fasting venous blood samples of radiation workers were collected in the morning and were placed in a centrifuge tube containing separation gel, centrifuged at 3000 rpm for 10 min, then collected serum samples were added in labeled test tubes, and test triiodothyronine (T3), thyroxine (T4), and thyroid-stimulating hormone (TSH) with automatic chemical immunoanalyzer (MAJLUMI4000PLUS, China), which are frequently used to evaluate thyroid function (41, 59).

#### 3.2.2 Liver and kidney function tests

Collected blood was centrifuged, following which the relevant biochemical blood index was measured using an automatic biochemical analyzer (Beckman, AU680, United States) (60), including the fasting blood glucose (FBG), creatinine, uric acid, blood urea nitrogen (BUN), alanine transaminase (ALT), gamma-glutamyl transferase (GGT), total bilirubin, direct bilirubin, alkaline phosphatase (ALP), aspartate transaminase (AST), etc.

#### 3.2.3 Chromosome aberration analysis

Chromosome aberrations are a biological dosimeter that can evaluate ionizing radiation injury in genetics (61). The process reference to the national standard of GBZ/T 248–2014 (62). Analyze the chromosome karyotype and interpret the test results as follows: (1)The normal reference range for the acentric fragment aberration rate is 0–3%; values exceeding 3% are considered abnormal (62) (2). A double centromere, centromeric ring, or stable chromosome aberration rate of ≥1% is deemed abnormal (62, 63) (3). Abnormal results may warrant retesting within 3–6 months (62).

#### 3.2.4 The micronucleus test

The micronucleus test (MNT) has been used as a screening method to demonstrate the cytoplasm’s aberrant chromosomal material (micronuclei). This test facilitates the rapid and automated detection of chromosome damage caused by radiation and indicates chromosomal instability (64). The process mainly references the national standard of GBZ/T 328–2023 (65). Finally, interpret the test results as follows: (1) The normal reference range for the micronucleus rate using the conventional culture method is 0–6%, while for the cytokinesis-block micronucleus assay, it ranges from 0 to 30% (63, 65). (2) If the test results exceed the normal reference range, chromosomal aberrations in peripheral blood lymphocytes should be further evaluated (65).

#### 3.2.5 Ophthalmic examinations

Routine ophthalmic examination includes visual acuity, color vision, vitreous, fundus, and a slit-lamp lens examination to investigate lens opacities, visual acuity (VA) in each eye was tested by using Snellen and Jaeger charts (66). Pupillary dilation to ≥6 mm is induced with 1% tropicamide, and digital photographs of the lens were obtained by using the slit lamp (SLM-KD4, China) (66), providing detailed information on the location and degree of opacity and confirming the cataract type (67).

#### 3.2.6 Urinalysis

Urinalysis was performed using an automated urine analyzer (URIT-1600, Guilin, China) for chemical and sediment analyses. Urine sediments were obtained by centrifugation at 500× g for 15 min, a drop of pellets was then placed on a glass slide, covered with a cover slip, and observed under a light microscope, proteinuria was evaluated semi-quantitative (+−/+/++/+++), and hematuria is defined as elevated urine erythrocyte quantification (>17/μL), as well as positive in semi-quantitative urinalysis (+−/+/++/+++) (68).

#### 3.2.7 Dermatological assessments

Radiation-induced skin injuries can be classified into acute or chronic injuries (69), and the grading criteria refer to GBZ-106-2020 (70). Acute injuries are characterized by symptoms such as erythema, depilation, burning sensation, numbness, itching, edema, and tingling. In contrast, chronic injuries manifest as skin pigmentation or loss, rough and gray nails, skin hyperkeratosis, chapping or atrophic thinning, telangiectasia, nail thickening and deformation, necrotic ulcer, etc. (70). The diagnosis and evaluation of radiation skin injuries are based on these methods: medical history and health examinations, including scenario details, dermatological assessments, timing and duration of signs and symptoms, digital color photographs, etc. (71).

#### 3.2.8 Audiometry

Hearing of radiation workers was evaluated through bilateral otoscopic (ITERA, Denmark) examinations conducted in a quiet, soundproof room. Hearing thresholds will be evaluated at 0.5, 1, 2, and 4 kHz frequencies to assess the degree of hearing loss, Hearing loss and its severity were assessed using pure tone threshold averages in the better ear. A pure-tone average exceeding 25 dB in the better ear indicates hearing loss, which is categorized as follows: slight hearing loss (>25 and ≤ 40 dB), moderate hearing loss (>40 and ≤ 60 dB), and severe hearing loss (>60 dB) (1, 2).

## 4 Discussion

This article outlines the study protocol for investigating occupational exposure among radiation workers in Chongqing, providing valuable insights and methodological strategies for cohort studies in the region. Despite the increasing awareness of chronic low-dose ionizing radiation exposure, the exposure-lag response associations with various adverse health outcomes remain underexplored, highlighting the need for further research in this area.

Some progress in the cross-sectional survey has been made in the occupational investigation of radiation workers with different health outcomes in China. Regarding the effects of occupational radiation exposure on the complete blood count, Zhang et al. (3) noted that low-dose ionizing radiation could increase the detection rate of abnormal white blood cells in radiation workers, yet no significant effect on hemoglobin and platelets. However, the influencing factors such as years of service and type of work were not further analyzed, which were closely related to the abnormal health outcome (4). Wang et al. showed that the occupational population had a higher risk of white blood cell and platelet counts, while no significant effect was observed on red blood cell counts (4). The cross-sectional survey design made it impossible to monitor the progression of health outcomes over an extended period. In contrast, a cohort study design incorporating longitudinal data enables the evaluation of temporal effects on health outcomes, facilitating the investigation of both long-term cumulative exposure-response relationships and exposure-lag-response dynamics.

Several cohort studies on occupational radiation exposure have been conducted in China, primarily examining outcomes such as cancer incidence and mortality (13, 72), changes in thyroid hormones (41) and complete blood count (73), liver damage (74), cataracts (66). A three-year cohort study showed that long-term low-dose radiation exposure was a risk factor for liver injury (74). In addition, a prospective cohort study explored the effects of occupational radiation exposure on platelets (54). Still, both studies exhibited certain limitations. Specifically, individual dose monitoring data were absent for previous years, and the cumulative radiation dose of the radiographers, as assessed using the job-exposure matrix (JEM) may have introduced measurement bias. Additionally, the radiation dose estimates were uncertain (54, 74). To further elucidate the health effects of occupational radiation exposure, two cohort studies were conducted over a five-year period, Liu et al. (54) performed a cohort involving 1265 medical workers exposed to low-dose ionizing radiation over the same duration. Their findings identified a cumulative exposure-response relationship, with an initial increase in platelet count followed by a subsequent decline (54). Additionally, a dose–response relationship was observed, whereby changes in platelet count were directly correlated with the cumulative dose of radiation received. Conversely, Wang et al. (73) found no significant differences in blood cell counts among 375 medical radiation workers engaged in different types of work. No statistical correlation has been found between the long-term cumulative exposure dose and the platelet count for a five-year period (73). The inconsistency in previous research findings may be attributed to short follow-up durations, limited cohort sizes, and a lack of focus on exposure-lag responses. These cohort studies primarily focus on the long-term cumulative exposure-response relationships, while overlooking the exposure-lag-response, which represents the complex interplay between health outcomes and the timing, duration, and intensity of exposure. This concept acknowledges that health effects from exposure may be delayed, with the risk of outcomes depending on both the intensity and timing of previous exposures.

To address these limitations, we have reviewed the characteristics of prior cohorts and are planning to establish a long-term, enough sample size, prospective cohort study in Chongqing to investigate the exposure-lag response associations and long-term cumulative exposure-response relationships between chronic low-dose ionizing radiation exposure and various adverse health outcomes. Notably, the average annual effective dose of radiation workers monitored in Chongqing from 2016 to 2020 remained below the national individual dose limit and demonstrated an overall downward trend (25, 26, 56).

This study has certain limitations. Data on the physical activity of radiation workers were not collected, which is a recognized risk factor for ocular trauma. Additionally, information on individual ultraviolet (UV) exposure, another significant factor when assessing cataract risk, was not included.

In summary, this study aims to evaluate the health effects of low-dose ionizing radiation exposure among radiation workers in Chongqing. Our study will provide more information on health outcomes and enhance the understanding of work practices and the exposure-lag response association between occupational radiation exposure and the health of radiation workers. It is recommended to further expand the sample size, extend the follow-up time, adopt more advanced statistical methods and improve the dosimetry method in the future, providing a reference for subsequent studies.
